# Comparative Evaluation of Five Multimodal Large Language Models for Medical Laboratory Image Recognition: Impact of Prompting Strategies on Diagnostic Accuracy

**DOI:** 10.3390/diagnostics16091258

**Published:** 2026-04-22

**Authors:** Hui-Ru Yang, Kuei-Ying Lin, Ping-Chang Lin, Jih-Jin Tsai, Po-Chih Chen

**Affiliations:** 1 Department of Laboratory Medicine, Kaohsiung Medical University Hospital, Kaohsiung 807, Taiwan; 2 Department of Medical Laboratory Science and Biotechnology, Kaohsiung Medical University, Kaohsiung 807, Taiwan; 3 Tropical Medicine Center, Kaohsiung Medical University Hospital, Kaohsiung 807, Taiwanjijits@cc.kmu.edu.tw (J.-J.T.); 4 School of Medicine, College of Medicine, Kaohsiung Medical University, Kaohsiung 807, Taiwan; 5 Division of Infectious Diseases, Department of Internal Medicine, Kaohsiung Medical University Hospital, Kaohsiung 807, Taiwan

**Keywords:** artificial intelligence, multimodal large language models, clinical laboratory, proficiency testing, image recognition, prompt engineering, blood smears, urinalysis, parasitology

## Abstract

**Background:** Multimodal large language models (MLLMs) show promise in medical imaging, but their performance is highly dependent on prompt engineering. This study systematically evaluates how different prompting strategies affect diagnostic accuracy in clinical laboratory image interpretation. **Methods:** We evaluated five MLLMs (ChatGPT-4o, Gemini 2.0 Flash, Claude 3.5 Sonnet, Grok-2, and Perplexity Pro (Claude 3.5 Sonnet)) using 177 proficiency testing images across three domains: blood smears (*n* = 78), urinalysis (*n* = 50), and parasitology (*n* = 49). Three prompting approaches were compared: (1) complex multi-choice prompts with 20 diagnostic options, (2) zero-shot open-ended prompts, and (3) two-step descriptive-reasoning prompts. Images were sourced from the Taiwan Society of Laboratory Medicine external quality assurance archives with expert consensus diagnoses. **Results:** Zero-shot prompting significantly outperformed complex multi-choice prompts across all models and domains (*p* < 0.001). With zero-shot prompts, Gemini achieved 78.5% overall accuracy (urinalysis: 92.0%; parasitology: 75.5%; blood smears: 64.1%), representing a 17% improvement over complex prompts. Two-step descriptive-reasoning prompts further improved blood smear accuracy by 8–12% for top-performing models, but showed minimal benefit in urinalysis and parasitology. The re-query mechanism (“please reconsider”) improved urinalysis accuracy by 7.6% but had a negligible effect on blood smears and parasitology. **Conclusions:** Prompting strategy critically determines MLLM diagnostic performance. Zero-shot approaches with minimal constraints consistently outperform complex multi-choice formats. The remarkable performance of general-purpose models in structured domains like urinalysis (>90% accuracy) demonstrates the considerable progress of multimodal AI. However, complex morphological tasks like blood smear interpretation require either specialized prompting techniques or domain-specific fine-tuning. These findings provide evidence-based guidance for optimizing AI integration in clinical laboratories.

## 1. Introduction

### 1.1. The Critical Role of Morphological Assessment in Clinical Laboratories

Clinical laboratory testing provides about 70% of the objective information used in clinical decision-making, diagnosis, and therapeutic monitoring [1]. Within this context, morphological assessment of biological specimens—including blood, urine, and tissue—continues to play an essential role in routine diagnostic practice. Accurate interpretation of clinical laboratory results relies heavily on the visual judgment and pattern recognition skills of medical technologists, particularly when identifying subtle cellular features, such as parasitic inclusions, or distinguishing malignant blast cells from reactive or benign lymphocytes.

Despite its diagnostic value, conventional microscopic examination has several inherent limitations, including high labor demand, time-consuming workflows, and inter-observer variability related to fatigue, inconsistent training, and subjective interpretation [2]. To address these challenges and to maintain analytical reliability, clinical laboratories participate in structured quality management systems, most commonly through external quality assurance (EQA) programs implemented via proficiency testing (PT) schemes [3,4]. In such programs, standardized unknown samples are periodically distributed to participating laboratories, and the reported results are compared via peer consensus to assess technical competence and diagnostic consistency.

With the continuous rise in global diagnostic testing, increasing attention has been directed toward automated approaches that may support human experts, improve the standardization of morphological interpretation, and reduce turnaround time for clinically critical results. As a result, digital pathology and artificial intelligence technologies have gradually been incorporated into clinical laboratory workflows.

### 1.2. Evolution of AI in Medical Image Analysis

The application of AI in medical imaging has evolved dramatically over the past decade. Traditional machine learning approaches, particularly convolutional neural networks (CNNs), have demonstrated strong performance in specific, well-defined classification tasks, such as reticulocyte counting and white blood cell differentiation, when trained on large, curated datasets [4,5]. Current commercial digital pathology systems leveraging CNN architectures achieve classification accuracies exceeding 95% for targeted applications and have been successfully integrated into laboratory workflows [6], demonstrating their clinical utility and reliability.

However, CNN-based systems typically require task-specific training and struggle to generalize across the diverse visual landscape of images acquired in clinical laboratories [5]. The recent emergence of multimodal large language models (MLLMs) represents a complementary paradigm. Unlike CNNs, these foundation models train on vast corpora of text and images, developing broad conceptual understanding across domains [5,7]. Theoretically, MLLMs can leverage encyclopedic medical knowledge to interpret novel images through zero-shot learning by synthesizing visual features with linguistic reasoning [7]. This flexibility may offer advantages in settings requiring rapid deployment, interpretability, or cost-effectiveness, particularly in resource-limited environments lacking specialized infrastructure.

Beyond laboratory medicine, MLLMs have been evaluated across a broad spectrum of medical domains. In radiology, vision–language models have demonstrated a capacity for automated report generation and image-finding summarization. In dermatology and ophthalmology, multimodal models have been applied to lesion classification and screening tasks, while in digital pathology, foundation models have shown promise for whole-slide image interpretation. The performance of these models in answering clinical questions and medical licensing examination have also been extensively benchmarked. These diverse applications underscore the growing interest in leveraging general-purpose AI for specialized medical tasks, while also highlighting the need for domain-specific evaluation frameworks to assess their real-world utility. A critical yet underexplored factor in MLLM performance is prompt engineering. Recent evidence suggests that diagnostic accuracy varies substantially based on how questions are framed [8]. Complex prompts may inadvertently constrain model reasoning, while zero-shot approaches and chain-of-thought techniques can unlock latent capabilities [9]. Therefore, understanding optimal prompting strategies is essential for reliable clinical deployment.

### 1.3. Research Objectives

While specialized AI software trained on massive datasets has aided in improving clinical workflows, it remains unclear whether general-purpose MLLMs can perform similarly without domain-specific fine-tuning. Previous studies suggest that MLLMs may demonstrate weaker performance in specialized visual recognition tasks compared to textual reasoning tasks. Therefore, a primary objective of this study was to verify whether these ‘off-the-shelf’ models could handle the rigor of proficiency testing. Previous studies have explored the capabilities of deep learning models, such as CNNs and YOLO architectures, in specific diagnostic tasks like blood cell classification, but comprehensive comparison across multiple models, prompting strategies, and diagnostic domains remains limited. This research involves a systematic evaluation of five leading MLLMs using a proficiency testing framework that mirrors real-world external quality assurance. The central question is as follows: Can general-purpose AI models, without specific fine-tuning, achieve clinically acceptable diagnostic accuracy in medical laboratory image interpretation, and to what extent does this depend on the prompting approach?

The aims of this study include the following: (1) benchmarking diagnostic accuracy using standardized, expert-validated PT images across blood smears, parasitology, and urinalysis; (2) systematically comparing complex multi-choice prompts, zero-shot prompts, and two-step descriptive-reasoning prompts to identify optimal strategies; (3) assessing iterative refinement capabilities to distinguish superficial retrieval errors from fundamental perceptual limitations [10]; and (4) providing evidence-based recommendations for AI integration into clinical laboratories. This benchmarking was conducted relative to expert consensus PT answers, not as a direct head-to-head performance comparison with individual humans.

## 2. Materials and Methods

### 2.1. Dataset and Ground Truth Establishment

The dataset comprised 177 high-resolution digital micrographs retrospectively collected from proficiency testing archives of the Taiwan Society of Laboratory Medicine (TSLM), Taiwan’s primary professional body for medical laboratory science, which provides accredited EQA services aligned with ISO/IEC 17043 standards [4,11]. The images represented diverse pathologies and normal variants encountered in routine clinical practice, classified into three primary categories:

- Blood smears (*n* = 78): This category included peripheral blood smears stained with Wright–Giemsa stain for identifying white blood cell lineages (neutrophils, lymphocytes, monocytes, eosinophils, basophils), immature precursors (blasts, myelocytes), red blood cell morphology (sickle cells, target cells, spherocytes), and platelet anomalies. For this domain, the task was defined as morphological classification of the primary cell or feature of interest, rather than clinical disease diagnosis. These preparations represent the most challenging category due to subtle morphological variations and overlapping features between cell types.
- Urinalysis (*n* = 50): This category included unstained wet-mount preparations of urine sediment for identifying epithelial cells, erythrocytes, leukocytes, casts (hyaline, granular, cellular), crystals (calcium oxalate, uric acid, triple phosphate), bacteria, and yeast.
- Parasitology (*n* = 49): This category included wet mounts and permanently stained preparations (trichrome, modified acid-fast) of stool or blood for identifying intestinal helminth eggs (*Ascaris*, *Trichuris*, hookworm), protozoan cysts and trophozoites (*Giardia*, *Entamoeba*, *Cryptosporidium*), and blood parasites (*Plasmodium* species, *Babesia*).

**Figure 1:**
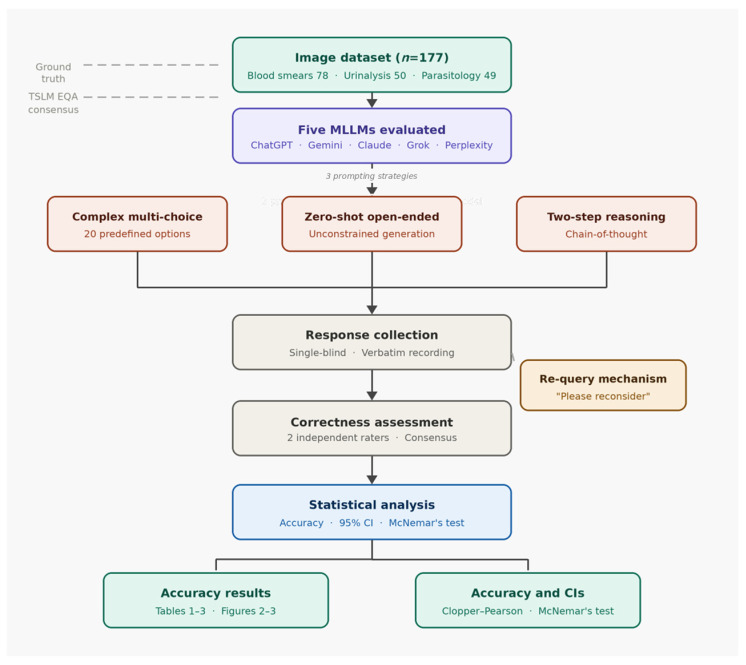
Study workflow diagram. Dashed lines indicate the sources of ground truth and external quality assurance consensus used in this study. EQA, external quality assurance; CI, confidence interval; TSLM, Taiwan Society of Laboratory Medicine.

The correct diagnosis for each image was established as the consensus answer of the TSLM expert committee during the original PT event, ensuring ground truth based on expert agreement rather than individual opinion. All images were completely de-identified and obtained from pre-existing standardized proficiency testing programs; therefore, this study qualifies as research on non-human subjects and is therefore exempt from ethical review by an institutional review board (IRB). To ensure unambiguous target identification, particularly in fields containing multiple cells or complex background elements, a visual indicator (e.g., an arrow) was digitally superimposed on each image to indicate the specific cell, organism, or structure to be evaluated. This step isolated the morphological classification task from object detection challenges. The overall study workflow is illustrated in Figure 1.

### 2.2. Multimodal Large Language Models Evaluated

Five commercially available MLLMs were evaluated through their publicly accessible interfaces during October–December 2024:

1. ChatGPT-4o (OpenAI): GPT-4 with vision capabilities, accessed via ChatGPT Plus subscription (interface version available October–December 2024; formal version string not disclosed by provider).
2. Gemini 2.0 Flash (Google): Google’s multimodal model, accessed via Google AI Studio (October–December 2024).
3. Claude 3.5 Sonnet (Anthropic): Anthropic’s vision-enabled model, accessed via Claude Pro (October–December 2024).
4. Grok-2 (xAI): xAI’s multimodal model, accessed via X Premium+ (October–December 2024).
5. Perplexity Pro: Perplexity’s AI search with vision capabilities (underlying language model: Claude 3.5 Sonnet; October–December 2024).

All evaluations were conducted using default interface settings without modifying the temperature, sampling parameters, or system prompts. Formal version strings were not always disclosed by the provider interfaces at the time of access, which inherently limits the reproducibility of the consumer-interface-based evaluation.

### 2.3. Experimental Design: Prompting Strategy Comparison

A critical innovation of this study is the systematic comparison of three prompting approaches, motivated by emerging evidence that prompt complexity may paradoxically impair performance [10,12]:

#### 2.3.1. Complex Multi-Choice Prompt (Initial Approach)

Images were initially queried using a structured prompt providing 20 diagnostic options corresponding to common findings in each category. For blood smears, the prompt was as follows: “Please analyze this blood smear image and select the most appropriate cell type from the following options: (1) Neutrophil, (2) Band neutrophil, (3) Metamyelocyte, (4) Myelocyte, (5) Promyelocyte, (6) Blast, (7) Lymphocyte, (8) Atypical lymphocyte, (9) Monocyte, (10) Eosinophil, (11) Basophil, (12) Plasma cell, (13) Nucleated RBC, (14) Target cell, (15) Sickle cell, (16) Spherocyte, (17) Schistocyte, (18) Platelet clump, (19) Platelet, (20) Artifact. Provide only the option number and name.” Similarly structured prompts with 20 domain-specific options were used for urinalysis and parasitology.

For urinalysis, the prompt was as follows: “Please analyze this urine sediment image and select the most appropriate finding from the following options: (1) Squamous epithelial cell, (2) Transitional epithelial cell, (3) Renal tubular epithelial cell, (4) Erythrocyte, (5) Leukocyte, (6) Hyaline cast, (7) Granular cast, (8) Waxy cast, (9) Cellular cast, (10) Calcium oxalate crystal, (11) Uric acid crystal, (12) Triple phosphate crystal, (13) Cystine crystal, (14) Bacteria, (15) Yeast, (16) Mucous thread, (17) Spermatozoa, (18) Artifact, (19) Trichomonas, (20) Other. Provide only the option number and name.”

For parasitology, the prompt was as follows: “Please analyze this parasitology image and select the most appropriate organism from the following options: (1) *Ascaris lumbricoides* egg, (2) *Trichuris trichiura* egg, (3) Hookworm egg, (4) *Enterobius vermicularis* egg, (5) *Giardia lamblia* cyst, (6) *Giardia lamblia* trophozoite, (7) *Entamoeba histolytica* cyst, (8) *Entamoeba histolytica* trophozoite, (9) *Cryptosporidium* oocyst, (10) *Blastocystis hominis*, (11) *Plasmodium falciparum*, (12) *Plasmodium vivax*, (13) *Babesia* spp., (14) *Taenia* egg, (15) *Opisthorchis*/*Clonorchis* egg, (16) *Schistosoma* egg, (17) *Strongyloides* larva, (18) *Microfilaria*, (19) Artifact, (20) Other. Provide only the option number and name.”

The 20 diagnostic options provided in each domain were derived directly from the answer categories used in the TSLM proficiency testing program, reflecting the actual classification framework employed in routine EQA assessment. No pilot optimization or iterative refinement of these prompts was performed prior to evaluation. This design reflects a realistic deployment scenario in which a laboratory professional structures a query using standard diagnostic terminology.

This approach, while seemingly logical, constrains the model to predefined categories and may inadvertently introduce noise through irrelevant options.

#### 2.3.2. Zero-Shot Open-Ended Prompt

Following initial results indicating suboptimal performance with complex prompts, we implemented a minimal zero-shot approach using the following prompt: “This is a [blood smear/urine sediment/parasitology] image from a clinical laboratory proficiency testing program. What is your diagnosis? Provide the specific cell type, organism, or structure visible in the image.”

This unbiased prompt allows models to leverage their full knowledge base without artificial constraints, consistent with emerging best practices in prompt engineering [13].

#### 2.3.3. Two-Step Descriptive-Reasoning Prompt

To evaluate whether explicit reasoning steps improve accuracy, we implemented a chain-of-thought approach [14] using the following prompt: “Step 1: Describe in detail the morphological features you observe in this image, including size, shape, color, internal structures, and any distinguishing characteristics. Step 2: Based on your description, what is your diagnosis? Explain your reasoning.”

This method forces explicit articulation of visual features before diagnosis, potentially improving accuracy through structured reasoning.

#### 2.3.4. Iterative Re-Query Mechanism

For all approaches, the following standardized re-query prompt was applied: “Please reconsider your answer carefully. Look again at the morphological details. What is your final diagnosis?” This tests whether models can self-correct initial errors through reflection [15].

### 2.4. Data Collection and Blinding

To simulate real-world PT conditions, each image was uploaded individually to each MLLM interface without providing diagnostic history or clinical context. A single-blind design was employed whereby investigators knew the ground truth diagnosis, but models received only the image and prompt. All queries were conducted by two independent investigators (H-RY, K-YL), with responses recorded verbatim. The expert consensus diagnosis from the original PT event served as the reference standard.

To control for temporal variations in model performance due to updates, all evaluations for a given prompting strategy were completed within a two-week window. Models were accessed through their standard consumer interfaces without API access or modification of default parameters.

Each image was submitted individually to each model interface without prior diagnostic context, and was queried once per prompting strategy per model. For the re-query condition, the standardized follow-up prompt was applied immediately after the initial response within the same conversation session. Output variability across repeated queries was not systematically assessed, as access was limited to consumer-facing interfaces without API-level control, which limits the study’s reproducibility.

### 2.5. Accuracy Assessment and Statistical Analysis

Diagnostic accuracy was calculated as the percentage of correct identifications (exact match with consensus diagnosis) for each model, domain, and prompting strategy. Binomial exact confidence intervals (95% CI) were computed using the Clopper–Pearson method, and the accuracy between prompting strategies was compared using McNemar’s test for paired proportions. Performance across domains and models was visualized using standard and horizontal bar charts. Statistical significance was defined as *p* < 0.05, and all analyses were performed using R version 4.3.1.

For zero-shot and two-step prompting conditions, model responses were in free-text format and correctness was assessed by two independent investigators (H-RY and K-YL), both trained medical technologists with clinical laboratory experience. A response was scored as correct if it contained the specific cell type, organism, or structure designated as the consensus PT answer, with allowance for standard synonyms and alternative nomenclature (e.g., “segmented neutrophil” accepted for “neutrophil”; “Giardia lamblia” and “Giardia intestinalis” both accepted). Ambiguous or partially correct responses were reviewed by both investigators and adjudicated by consensus discussion. Formal inter-rater reliability assessment was not performed, as scoring was conducted through a collaborative consensus process rather than independent parallel rating.

## 3. Results

### 3.1. Impact of Prompting Strategy on Diagnostic Accuracy

To establish a performance baseline, the dataset was evaluated by licensed medical technologists, who consistently achieved near-perfect scores (close to 100%). This human benchmark highlights the gap between current AI capabilities and professional competency. Zero-shot open-ended prompts significantly outperformed complex multi-choice prompts across all five models and all three diagnostic domains (*p* < 0.001). Table 1 presents the comparative accuracy results. With zero-shot prompts, Gemini achieved the highest overall accuracy (78.5%, 95% CI: 72.0–84.0%), representing a 17% improvement over the initial complex-prompt approach (67.2%), and ChatGPT showed similar improvements (72.3% vs. 60.5%, +11.8%). This pattern was consistent across all models, with improvements ranging from 8.4% (Grok) to 17.0% (Gemini).

The performance gap between prompting strategies was most pronounced in blood smear analysis, where zero-shot prompts yielded accuracy improvements of 10–15% for top-performing models. In urinalysis, where structured geometric features predominate, the advantage of zero-shot prompts was less apparent but still significant (4–6% improvement). Parasitology showed intermediate results, with zero-shot approaches resulting in improvements of 7–10%.

Values are presented as the percentages with 95% confidence intervals (Clopper–Pearson method). The following zero-shot prompt was used: “This is a [domain] image from a clinical laboratory proficiency testing program. What is your diagnosis?” All models showed the highest accuracy in urinalysis and the lowest in blood smears.

### 3.2. Domain-Specific Performance Patterns

**Figure 2:**
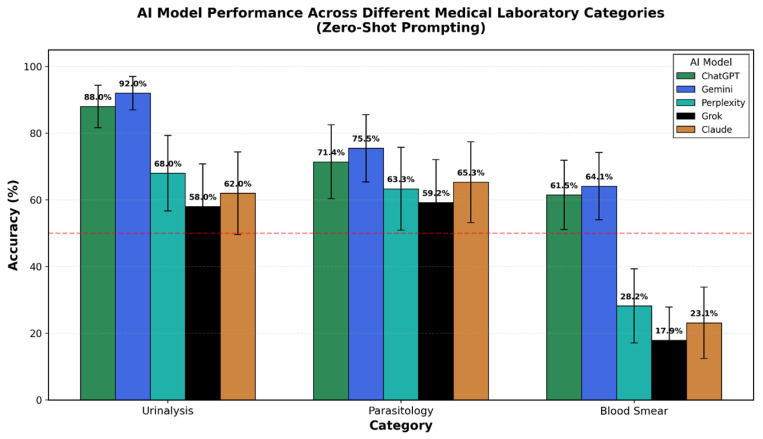
Comparative diagnostic accuracy of five multimodal large language models across medical laboratory categories using zero-shot prompts. Bar chart showing overall accuracy and domain-specific performance for blood smears, parasitology, and urinalysis. Gemini (78.5%) and ChatGPT (72.3%) demonstrated superior overall performance. All models showed the highest accuracy in urinalysis (Gemini: 92.0%, ChatGPT: 88.0%) and the lowest in blood smears (Gemini: 64.1%, ChatGPT: 61.5%). Error bars represent 95% confidence intervals calculated using the Clopper–Pearson method. The red dashed line indicates the 50% accuracy threshold.

Across all prompting strategies, urinalysis consistently yielded the highest accuracy, followed by parasitology, with blood smears presenting the greatest challenge. Using optimal zero-shot prompts, Gemini achieved 92.0% accuracy in urinalysis (95% CI: 81.2–97.0%), 75.5% in parasitology (95% CI: 61.7–85.6%), and 64.1% in blood smears (95% CI: 52.8–74.2%). ChatGPT demonstrated comparable performance with 88.0%, 71.4%, and 61.5% accuracy, respectively (Figure 2).

The three lower-performing models (Perplexity, Claude, Grok) showed greater performance variability. While they achieved respectable accuracy in urinalysis (62–72%), their performance in blood smears was substantially lower (18–28%), suggesting fundamental differences in visual processing capabilities or training data composition.

The performance patterns across domains indicate that the diagnostic challenge is not uniformly distributed, but rather, domain-specific, with morphologically complex tasks like cytology proving substantially more difficult than geometric structure recognition. Urinalysis showed uniformly higher accuracy across models, contrasting sharply with blood smears, particularly for Claude and Grok.

### 3.3. Two-Step Descriptive-Reasoning Prompts

The two-step chain-of-thought approach demonstrated selective benefits. In blood smear analysis, it improved accuracy by 8–12% for Gemini and ChatGPT compared to standard zero-shot prompts (Gemini: 64.1% to 72.4%, ChatGPT: 61.5% to 70.5%, *p* < 0.01). The forced-description step appeared to help models focus on critical morphological details such as the nuclear–cytoplasmic ratio, chromatin pattern, and cytoplasmic granulation.

However, two-step prompts showed minimal benefit in urinalysis and parasitology (<2% improvement), and actually decreased performance to lower levels than those of lower-tier models, possibly due to error propagation, where incorrect initial descriptions constrained subsequent reasoning. This suggests that chain-of-thought prompting is most valuable when visual features are subtle and require explicit attention, but may be unnecessary or even detrimental for more straightforward recognition tasks.

### 3.4. Self-Correction Through Iterative Re-Querying

**Figure 3:**
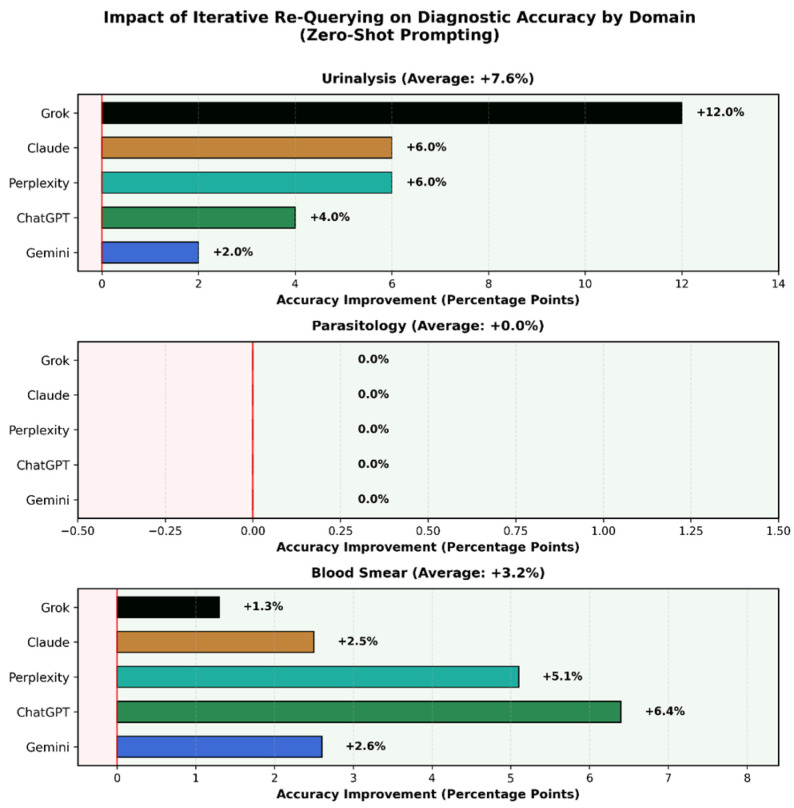
Impact of iterative re-querying on diagnostic accuracy using zero-shot prompts by domain. Three-panel horizontal bar chart displaying accuracy improvement (percentage points) after the “Please reconsider” prompt for each model across the urinalysis, parasitology, and blood smear categories. Urinalysis demonstrates significant self-correction capability, with an average improvement of +7.6% across all models (range: +2.0% to +12.0%), with Grok showing the largest gain. Parasitology shows complete absence of self-correction (0.0% improvement for all models), indicating fundamental perceptual limitations rather than correctable reasoning errors. Blood smears show modest improvement (+3.2% average, range: +1.3% to +6.4%), suggesting intermediate error mechanisms. This domain-dependent pattern reveals that errors in structured geometric tasks (urinalysis) are amenable to reflective correction, while errors in complex morphological interpretation (parasitology, blood cytology) stem from insufficient visual feature extraction that cannot be overcome through iterative prompting alone. Bar colors represent individual models: Gemini (dark blue), ChatGPT (dark green), Perplexity (dark turquoise), Claude (brown), and Grok (black).

The standardized re-query mechanism (“please reconsider”) showed domain-dependent effects (Table 2, Figure 3). In urinalysis, re-querying improved accuracy by an average of 7.6% across models (range: 2.0–12.0%, *p* < 0.01), with Gemini improving from 88.0% to 90.0%, and ChatGPT exhibiting the greatest improvement from 74.0% to 84.0%. This suggests that initial urinalysis errors often represent superficial retrieval mistakes or hasty pattern matching that can be corrected through reflection.

In contrast, parasitology showed no improvement following re-querying (0.0% average change), and blood smears demonstrated minimal improvement (3.2% average). The lack of self-correction in these domains indicates that errors stem from fundamental perceptual limitations rather than correctable reasoning mistakes. Models that initially misidentify complex morphological features rarely correct themselves, suggesting that their visual representations lack the necessary discriminative information. The complete accuracy data across all three prompting strategies and domains are presented in Table 3.

This differential response to re-querying provides insight into error mechanisms. In structured domains like urinalysis with distinctive geometric features (cast shapes, crystal morphology), models possess adequate visual information but may make linguistic or reasoning errors. In cytomorphology, errors more often reflect insufficient visual feature extraction.

The re-query prompt was as follows: “Please reconsider your answer carefully. Look again at the morphological details. What is your final diagnosis?” Urinalysis showed significant improvement (+7.6% average), parasitology showed no improvement (0.0% average), and blood smears showed minimal improvement (+3.2% average). This pattern suggests domain-dependent error mechanisms.

The values are presented as accuracy (%) with 95% confidence intervals (Clopper–Pearson method). Denominators: Blood smears *n* = 78, Urinalysis *n* = 50, Parasitology *n* = 49, Overall *n* = 177. Zero-shot prompts significantly outperformed complex multi-choice prompts across all models and domains (*p* < 0.001, McNemar’s test). Two-step reasoning provided selective additional benefits in blood smear analysis for top-performing models.

## 4. Discussion

The goal of this study was not to compare AI performance directly against individual human expert judgment, but rather, to characterize how prompting strategy influences model accuracy within a clinically grounded proficiency testing framework—and to identify conditions under which general-purpose MLLMs may offer reliable decision support. This study shows that diagnostic performance of multimodal large language models in clinical laboratory image interpretation is strongly influenced by the choice of prompting strategy. In particular, zero-shot open-ended prompts yielded higher accuracy than complex multi-choice formats across all evaluated models, indicating that increased structural constraint does not necessarily translate into improved performance. Notably, in the domain of urinalysis, several general-purpose models achieved accuracy exceeding 90% despite the absence of domain-specific training, suggesting that foundation models are capable of reliable performance in structured morphological tasks.

### 4.1. The Prompt Engineering Paradox

The observation that simple zero-shot prompts outperform complex multi-choice formats is consistent with recent work in prompt engineering suggesting that multimodal large language models can, in some cases, generate effective task framings without an extensive human-imposed structure [12,16]. Prior studies in the field of computer vision have reported improved performance with minimal or unbiased prompting strategies, including so-called “zero-instruction vision” approaches [16]. Our results extend these observations to a clinical laboratory context, indicating that similar principles apply to medical image interpretation tasks.

One possible explanation is that highly structured prompts may inadvertently restrict model behavior. When a fixed set of predefined options is provided, the model may be forced to consider diagnostically irrelevant alternatives, increasing cognitive noise. In addition, excessive prompt complexity may alter how visual and textual information is weighted during inference, potentially diverting attention from salient morphological features. Together, these factors may limit the model’s ability to fully exploit its underlying representational capacity.

From a practical perspective, these findings suggest that laboratories adopting AI-assisted diagnostic tools should consider favoring concise, open-ended prompts over heavily structured formats. Although further validation is required, this approach may also be applicable to other medical imaging domains that rely on pattern recognition rather than rigid categorical classification.

It should be noted that the comparison between complex multi-choice and zero-shot prompts is not purely intended to assess prompting strategy manipulation; the two formats also differ in terms of task structure and response generation constraints. The multi-choice format requires selection from a fixed option set, while the zero-shot format requires unconstrained generation. These differences reflect distinct real-world deployment scenarios, and the observed accuracy differences should be interpreted as reflecting the combined effect of prompt structure and task format. Future studies employing fully controlled prompt-format experiments—holding task type constant while varying prompt complexity—would help isolate the independent contribution of each factor.

### 4.2. Chain-of-Thought Reasoning in Visual Diagnostics

The observation that two-step descriptive-reasoning prompts provided additional benefits in blood cytology is consistent with prior work indicating that explicit reasoning steps can improve performance in complex tasks [14]. In this setting, requiring the model to describe morphological features before assigning a diagnosis may help it to focus on diagnostically relevant visual details throughout the reasoning process.

This effect was not uniform across diagnostic domains. In urinalysis, where key features are often defined by relatively distinct geometric patterns, such as cast morphology or crystal shape, direct visual pattern matching appeared to be sufficient. In contrast, blood smear interpretation involves integrating multiple subtle features, including nuclear chromatin structure, cytoplasmic characteristics, and overall cell architecture, which may benefit from a more explicit stepwise reasoning process.

Together, these results indicate that chain-of-thought prompting is most useful when applied to visually complex diagnostic tasks, rather than as a general strategy across all domains. Therefore, it may be important to consider task-specific morphological complexity when selecting prompting approaches for clinical image interpretation.

### 4.3. Error Mechanisms and Self-Correction Capabilities

Differences in model behavior following re-querying were observed across diagnostic domains, indicating distinct sources of error. In urinalysis, the presence of measurable self-correction suggests that many initial mistakes were not due to a lack of visual information, but rather, to premature pattern matching or incorrect label selection. In these cases, the model appeared capable of revising its initial response when prompted to re-examine the image, consistent with the idea that errors arise at the decision or retrieval stage rather than from perceptual failure [17].

In contrast, little to no self-correction was observed in parasitology, and only minimal improvement was seen in blood smear interpretation. When errors occurred in these domains, models rarely revised their answers, particularly in cases involving helminth egg identification or white blood cell lineage classification. This pattern suggests that the underlying visual representations were insufficient to support accurate discrimination, a limitation that cannot be readily corrected through iterative prompting alone. Similar phenomena have been described in prior studies of hallucination in language and vision–language models, where confident but incorrect outputs are associated with inadequate grounding in the input data [17].

From a practical standpoint, these observations indicate that different verification strategies may be required depending on the diagnostic task. In urinalysis, automated re-checking or secondary prompting steps may help recover a meaningful proportion of errors. For cytomorphological tasks, however, approaches such as ensemble modeling or domain-specific fine-tuning are more likely to be necessary to address underlying perceptual constraints.

The persistently lower accuracy in blood smear interpretation likely reflects a combination of interacting factors. First, fine-grained hematological morphology involves subtle distinctions—nuclear chromatin texture, cytoplasmic granularity, nuclear-to-cytoplasmic ratio—that require high discriminative visual resolution, which is unlikely to be well encoded in general-purpose models trained predominantly on internet-sourced data [5]. Second, training data distribution is a plausible contributor: high-magnification clinical cytology images are underrepresented in general corpora relative to their diagnostic importance. Third, categorical ambiguity is intrinsically high in blood cell classification; identifying the boundaries between, for example, reactive and atypical lymphocytes is challenging even for experienced morphologists [18]. Disentangling these factors will require future studies using controlled image perturbation, attention visualization, or fine-tuned model comparisons.

### 4.4. Task Decomposition and Ecological Validity

An important methodological consideration in interpreting the results of this study is the use of digitally superimposed arrows to indicate the specific cell, organism, or structure to be classified. This approach was intentionally employed to isolate morphological classification performance from object detection and scanning behaviors, thereby providing a controlled and reproducible evaluation framework. However, this task decomposition diverges from routine clinical microscopy, in which medical technologists must scan multiple fields, identify relevant structures among a heterogeneous cellular background, and integrate morphological findings with clinical context. Accordingly, the accuracy values reported here reflect classification under idealized conditions and should not be directly extrapolated to full-workflow clinical performance. Statements suggesting that the performance of foundation models may approach that of specialist-level systems should be interpreted with this constraint in mind. Future studies incorporating full-field image interpretation and multi-step clinical reasoning tasks will be necessary to evaluate the true translational potential of these models.

### 4.5. The Progress of Foundation Models

When interpreted in the context of recent advances in artificial intelligence, the results of this study highlight the increasing capability of general-purpose multimodal models in selected diagnostic tasks. In structured domains such as urinalysis, several models achieved accuracy exceeding 90%, despite the absence of training specifically tailored to clinical laboratory diagnostics. Under these conditions, the performance of foundation models approached, and in some cases approximated, that of specialized convolutional neural network-based systems.

These observations are consistent with a broader trend in AI development toward more flexible, general-purpose architecture. Compared with narrowly optimized task-specific models, foundation models offer potential advantages in terms of adaptability and interpretability, particularly in diagnostic scenarios where training data are limited or task definitions are less rigid. However, such advantages should be considered in relation to the specific performance requirements and constraints of each application [8,19,20].

Importantly, the findings of this study do not suggest that multimodal large language models can replace established clinical AI platforms. Rather, they support a complementary role in which general-purpose models may be well suited for structured or knowledge-intensive diagnostic tasks, while the existing CNN-based systems remain more effective for high-throughput routine analyses, such as standard differential counts. Hybrid strategies that integrate the strengths of both approaches may therefore represent a practical direction for future clinical implementation.

### 4.6. Integration with the Current Literature

Our findings align with and expand upon several recent studies. Using 70 EQA images from the Chinese National Center for Clinical Laboratories, Cai et al. [18] demonstrated that GPT-4O achieved 70% overall accuracy in blood cell morphology recognition (77.14% for peripheral blood smears, 62.86% for bone marrow smears), significantly lower than the accuracy of hematologists (95.42%). In line with our results, they concluded that GPT-4O’s performance was inadequate for clinical use without systematic comparison of prompting strategies. In the domain of urinalysis, Alizadeh et al. developed a multi-head YOLOv12 architecture with self-supervised pretraining for urinary sediment particle detection. The model was pretrained on 5640 unlabeled images and fine-tuned on 790 annotated images (containing 31,285 bounding boxes across 39 categories), achieving a precision of 76.59% (±3.29%) and a mean average precision (mAP) of 64.15% (±1.56%). Clinical validation on 84 patients demonstrated a mean accuracy of 86.55%, with performance ranging from 59.36% for bacteria to 97.10% for epithelial cells. However, significant limitations necessitate human verification. For discrepant samples, independent expert review remains essential. The authors indicated that the system should be positioned as a screening and decision-support tool rather than a fully autonomous diagnostic system, requiring human oversight for bacterial detection, rare particles, morphologically similar cells, and low-confidence predictions. The authors of [21] demonstrated that the performance of advanced vision–language models can match or exceed that of specialists in complex radiology tasks, such as report generation, when utilizing optimized prompting and fine-tuning strategies. This directly supports our conclusion that prompt engineering is critical for maximizing model utility.

The theoretical framework of chain-of-thought prompting, initially developed for text-only tasks, has shown promise in multimodal contexts [10]. Our domain-specific results refine this understanding, suggesting that the benefits of chain-of-thought prompting scale with task complexity rather than applying uniformly.

The superior performance of zero-shot prompts over complex prompts corroborates recent computer vision research showing that minimal instruction often outperforms elaborate task framing [12]. Our study provides clinical validation of these principles with direct relevance to medical AI implementation.

Recent advances in parameter-efficient adaptation of foundation models—including low-rank adaptation (LoRA) strategies and structured multimodal alignment frameworks—suggest that there is potential to substantially improve their domain-specific performance in biomedical tasks [22]. These approaches represent a complementary pathway to prompt optimization and are likely to be important for achieving specialist-level accuracy in complex morphological tasks. However, they require labeled training data, computational infrastructure, and ongoing maintenance, which may limit accessibility in many clinical laboratory settings. The present study addresses a more immediately deployable solution: optimizing inference-time behavior through prompt design without modifying model weights.

### 4.7. Clinical Implications and Regulatory Considerations

From a clinical laboratory perspective, the present findings have several practical implications for the use of multimodal large language models. Among the evaluated domains, urinalysis appears to represent the most immediately applicable use case. With optimal prompting, top-performing models achieved accuracy exceeding 90%, suggesting that these systems may be suitable as decision-support tools for tasks such as flagging atypical findings or providing differential considerations. Nevertheless, obtaining regulatory approval and validation in prospective clinical studies remains essential before such applications can be considered for routine use.

In contrast, blood smear interpretation warrants a more cautious approach. Although model performance improved with optimized prompting strategies, accuracy remained below the levels required for autonomous clinical decision-making. In this context, general-purpose models may be more appropriately positioned as adjunct tools for educational purposes, quality assurance activities, or preliminary screening, provided that all flagged cases undergo mandatory human review. This limitation distinguishes current multimodal models from established domain-specific AI systems, such as CellaVision, which have been specifically trained and extensively validated for clinical cytomorphological analysis. CellaVision is currently one of the most commonly used automated systems for blood cell analysis in clinical labs. It performs reasonably well with routine cells like neutrophils (96.46%) and lymphocytes (95.96%), and catches target cells with 100% sensitivity. However, the system struggles with clinically important abnormalities: basophil and eosinophil identification accuracy drops to 62–66%, platelet clumps are missed about 17% of the time, schistocytes are detected in only 78% of cases, and blast cell counts show poor agreement with manual counts (R^2^ = 0.57) until an expert reviews and corrects them. The message is clear—even mature, FDA-approved technology that has been employed in labs for years cannot replace skilled morphologists when it matters most [23].

Beyond task-specific performance, broader implementation challenges must also be considered. Frequent model updates and limited transparency in underlying architectures complicate conventional validation and certification processes, highlighting the need for regulatory approaches that can accommodate continuously evolving AI systems while ensuring patient safety.

Finally, cost-effectiveness considerations are likely to influence adoption decisions. Although foundation models reduce the need for large, task-specific training datasets and dedicated infrastructure, their subscription costs and computational demands must be weighed against their potential benefits. This is particularly relevant for high-volume routine testing, where specialized CNN-based systems may continue to offer greater efficiency.

### 4.8. Limitations

This study has several limitations that should be acknowledged. First, models were evaluated through publicly available consumer interfaces rather than through direct API access. As a result, reproducibility may be affected as model versions continue to evolve. Nevertheless, this approach reflects real-world usage conditions in many clinical laboratories, where access is often limited to consumer-facing platforms.

Second, the number of images in each diagnostic category was relatively modest (*n* = 49–78), which may have reduced the model’s statistical power for detecting more subtle differences in performance. In addition, diagnostic accuracy was assessed at the level of individual cells or structures, without evaluation of full-field image interpretation or integration of findings across multiple microscopic fields, a process that more closely resembles routine clinical workflow.

The dataset itself also warrants consideration. All images were derived from proficiency testing materials selected primarily for educational purposes, which may overrepresent classic or well-defined presentations compared with the ambiguous or borderline cases frequently encountered in daily practice. Furthermore, this study did not examine the impact of image quality issues, such as staining variability, artifacts, or technical imperfections, which can substantially complicate real-world morphological interpretation.

Finally, only five multimodal models were included in the present analysis. Given the rapid pace of development in this field, newer architectures may exhibit different performance characteristics. Ongoing and repeated evaluation will therefore be necessary to ensure that conclusions remain applicable as multimodal AI technologies continue to evolve.

A potential concern in any evaluation of MLLMs using external benchmark images is the possibility of training data contamination—i.e., that evaluation images may have been included in model training corpora. Although the PT images used in this study are derived from a controlled EQA archive not publicly available on the internet, we cannot formally exclude this possibility. Future studies should consider using prospectively collected images or time-stamped data specifically generated after the knowledge cutoff dates of the models under evaluation.

Furthermore, per-category classification records were not systematically retained across all prompting conditions, precluding post hoc computation of macro-averaged precision, recall, and F1-scores. Future studies should prospectively record per-category response data to enable full multi-class performance reporting.

## 5. Conclusions

This study provides a systematic evaluation of how prompting strategies influence the diagnostic performance of multimodal large language models in clinical laboratory image interpretation. Across all evaluated domains, zero-shot open-ended prompts consistently yielded higher accuracy than complex structured formats, underscoring the critical role of prompt design in determining model performance.

Performance varied substantially by diagnostic task. In urinalysis, general-purpose foundation models achieved accuracy exceeding 90% without domain-specific training, indicating that these models can perform reliably in structured morphological contexts. In contrast, tasks involving complex cytomorphological interpretation, such as blood smear analysis, remained challenging. In these settings, performance improvements were observed only with more sophisticated prompting strategies, such as chain-of-thought reasoning, or through domain-specific optimization, suggesting that there are inherent limitations in current general-purpose models.

The domain-dependent effects of iterative re-querying further highlight distinct error mechanisms. While models demonstrated the capacity for self-correction in urinalysis, likely reflecting recoverable decision-level errors, similar approaches provided limited benefits in cytomorphological tasks, where perceptual constraints appear to play a more prominent role.

Together, these findings indicate that multimodal large language models could play a complementary role in clinical laboratory diagnostics. Rather than replacing existing domain-specific systems, general-purpose models may offer added value in selected contexts that benefit from flexible reasoning and broad knowledge integration. As the performance of LLMs in medical imaging continues to mature, their integration into clinical diagnostic workflows—not as replacements, but as intelligent assistants—seems increasingly feasible, and may occur sooner than anticipated.

## Figures and Tables

**Table 1:** Comparative diagnostic accuracy with zero-shot prompts.

AI Model	Overall (*n* = 177)	Blood Smears (*n* = 78)	Parasitology (*n* = 49)	Urinalysis (*n* = 50)
Gemini	78.5% (72.0–84.0%)	64.1% (52.8–74.2%)	75.5% (61.7–85.6%)	92.0% (81.2–97.0%)
ChatGPT	72.3% (65.2–78.5%)	61.5% (50.1–71.9%)	71.4% (57.4–82.5%)	88.0% (76.2–94.4%)
Perplexity	49.2% (41.8–56.6%)	28.2% (19.0–39.3%)	63.3% (48.9–75.7%)	68.0% (54.2–79.3%)
Claude	45.8% (38.5–53.2%)	23.1% (14.9–33.8%)	65.3% (51.0–77.4%)	62.0% (47.9–74.4%)
Grok	41.2% (34.1–48.7%)	17.9% (10.8–27.9%)	59.2% (44.9–72.1%)	58.0% (44.1–70.8%)

**Table 2:** Effect of iterative re-query prompting with zero-shot approach.

Model	Blood Initial	Blood Re-Query	Parasite Initial	Parasite Re-Query	Urine Initial	Urine Re-Query
Gemini	64.1%	66.7%	75.5%	75.5%	92.0%	94.0%
ChatGPT	61.5%	67.9%	71.4%	71.4%	88.0%	92.0%
Perplexity	28.2%	33.3%	63.3%	63.3%	68.0%	74.0%
Claude	23.1%	25.6%	65.3%	65.3%	62.0%	68.0%
Grok	17.9%	19.2%	59.2%	59.2%	58.0%	70.0%

**Table 3:** Comparative diagnostic accuracy across three prompting strategies.

Model	Complex Multi-Choice				Zero-Shot Open-Ended				Two-Step Reasoning			
	Blood Smears (*n* = 78)	Urinalysis (*n* = 50)	Parasitology (*n* = 49)	Overall (*n* = 177)	Blood Smears (*n* = 78)	Urinalysis (*n* = 50)	Parasitology (*n* = 49)	Overall (*n* = 177)	Blood Smears (*n* = 78)	Urinalysis (*n* = 50)	Parasitology (*n* = 49)	Overall (*n* = 177)
Gemini	53.8% (42.9–64.5%)	86% (73.8–93%)	67.3% (53.4–78.8%)	66.7% (59.4–73.2%)	64.1% (53–73.9%)	92% (81.2–96.8%)	75.5% (61.9–85.4%)	78.5% (71.9–83.9%)	53.8% (42.9–64.5%)	84% (71.5–91.7%)	65.3% (51.3–77.1%)	65.5% (58.3–72.1%)
ChatGPT	47.4% (36.7–58.4%)	64% (50.1–75.9%)	57.1% (43.3–70%)	54.8% (47.4–62%)	61.5% (50.4–71.6%)	88% (76.2–94.4%)	71.4% (57.6–82.2%)	72.3% (65.3–78.4%)	46.2% (35.5–57.1%)	82% (69.2–90.2%)	67.3% (53.4–78.8%)	62.1% (54.8–69%)
Perplexity	9% (4.4–17.4%)	54% (40.4–67%)	55.1% (41.3–68.1%)	34.5% (27.9–41.7%)	28.2% (19.4–39%)	68% (54.2–79.2%)	63.3% (49.3–75.3%)	49.2% (41.9–56.5%)	17.9% (11–27.9%)	60% (46.2–72.4%)	65.3% (51.3–77.1%)	42.9% (35.9–50.3%)
Claude	11.5% (6.2–20.5%)	46% (33–59.6%)	57.1% (43.3–70%)	33.9% (27.3–41.1%)	23.1% (15.1–33.6%)	62% (48.2–74.1%)	65.3% (51.3–77.1%)	45.8% (38.6–53.1%)	11.5% (6.2–20.5%)	56% (42.3–68.8%)	63.3% (49.3–75.3%)	38.4% (31.6–45.8%)
Grok	10.3% (5.3–19%)	36% (24.1–49.9%)	51% (37.5–64.4%)	28.8% (22.6–35.9%)	17.9% (11–27.9%)	58% (44.2–70.6%)	59.2% (45.2–71.8%)	41.2% (34.3–48.6%)	10.3% (5.3–19%)	48% (34.8–61.5%)	49% (35.6–62.5%)	31.6% (25.2–38.8%)

## Data Availability

The original contributions presented in this study are included in the article. Further inquiries can be directed to the corresponding author.
